# Single-dose pharmacokinetics, safety, and tolerability of the dual endothelin receptor antagonist aprocitentan in subjects with moderate hepatic impairment

**DOI:** 10.1038/s41598-022-22470-z

**Published:** 2022-11-09

**Authors:** Magda S. C. Fontes, Jasper Dingemanse, Atef Halabi, Monika Tomaszewska-Kiecana, Patricia N. Sidharta

**Affiliations:** 1 Department of Clinical Pharmacology, Idorsia Pharmaceuticals Ltd, Hegenheimermattweg 91, 4123 Allschwil, Switzerland; 2 Clinical Research Services Kiel GmbH, Kiel, Germany; 3 Biokinetica S.A., Józefów, Poland

**Keywords:** Phase I trials, Medical research, Drug development

## Abstract

The effect of moderate hepatic impairment on the pharmacokinetics (PK), safety, and tolerability of the dual endothelin receptor antagonist aprocitentan was clinically investigated as 25% of aprocitentan is cleared through the liver. Aprocitentan is in clinical development for the treatment of resistant hypertension. This was an open-label, Phase 1 study. Subjects were recruited in two groups (i.e., moderate hepatic impairment (Child–Pugh B; n = 8) and matched healthy subjects (n = 9) and received a single oral dose of 25 mg aprocitentan. Thereafter, they were observed for 14 days. Due to personal reasons one healthy subject discontinued the study. The PK of aprocitentan were similar between subjects with moderate hepatic impairment and healthy subjects, with maximum plasma concentrations (C_max_) reached at 4.0 h. There was no difference in C_max_, indicated by the geometric means ratio (90% confidence interval) of 1.03 (0.86–1.24). There was a lower apparent clearance, a similar apparent volume of distribution, a longer terminal half-life (56.4 h vs 48.3 h in healthy subjects), and an increase in area under the curve from zero to infinity of 23% in moderate hepatically impaired subjects compared to healthy subjects. There were no differences observed in plasma protein binding (range 98.7–99.0%). Aprocitentan was well tolerated, and headache was the only adverse event reported by one subject. In conclusion, there were no clinically relevant differences in PK between subjects with moderate hepatic impairment and healthy subjects. Based on these results, aprocitentan can be administered in subjects with mild and moderate hepatic impairment and dose adjustment is not required.

*Clinical Trial Registration* ClinicalTrials.gov NCT04252495.

## Introduction

Endothelin (ET)-1, a 21-amino acid peptide mainly produced by endothelial cells, is one of the most potent vasoconstrictors known^1^. ET-1 and its two receptors, ET_A_ and ET_B_, which are widely distributed on many cell types, including cardiac, renal, and vascular cells, mediate biological processes that contribute to the pathogenesis of hypertension. In addition, ET-1 can also cause endothelial dysfunction, increased aldosterone synthesis and secretion, neurohormonal and sympathetic activation, and vascular hypertrophy and remodeling^2–4^. Blockage of ET-1 receptors has demonstrated efficacy in different models of hypertension, especially in low-renin/salt-sensitive conditions^5,6^.

Aprocitentan is an orally active, once daily, dual endothelin (ET_A_/ET_B_) receptor antagonist (ERA) currently in clinical development for the treatment of resistant hypertension. Aprocitentan has been investigated in several clinical Phase 1 studies. In an 8 weeks Phase 2 study in patients with essential hypertension, aprocitentan dose-dependently reduced sitting systolic and diastolic blood pressure at doses ranging from 10 to 50 mg once daily^7^. A clinical Phase 3 study in patients with resistant hypertension was recently completed with doses of 12.5 and 25 mg aprocitentan (ClinicalTrials.gov: NCT03541174). Results showed that aprocitentan significantly reduced blood pressure when added to standardized combination background antihypertensive therapy in patients with resistant hypertension over 48 weeks of treatment^8^.

In healthy subjects, aprocitentan was well tolerated at single oral doses of up to and including 600 mg, and at multiple oral doses of up to and including 100 mg once daily for 10 days^9^. After absorption of aprocitentan, with maximum plasma concentration (C_max_) reached at 3–9 h for the different doses, elimination was slow, with a terminal half-life (t_½_) of approximately 44 h^9^. Based on pharmacokinetic (PK) evaluations, aprocitentan can be administered with or without food and no dose adjustment is required for sex, age, ethnicity, or renal function impairment^9–11^.

Investigation of the absorption, distribution, metabolism, and excretion of aprocitentan in healthy subjects indicated that aprocitentan and its metabolites were eliminated by renal and hepatic routes, with urine (52% of total excretion) representing a more important elimination route than feces (25% of total excretion)^12^. Unchanged aprocitentan represented 0.2% and 6.8% of the administered dose excreted in urine and feces, respectively. In addition, unchanged aprocitentan was detected nearly exclusively in plasma, excluding the presence of relevant circulating metabolites. Formation of the two most abundant metabolites of aprocitentan M3 (excreted 24% in urine and 1% in feces) and M1 (excreted 2% in urine and 10% in feces) was catalyzed by uridine 5′-diphospho-glucuronosyltransferases 1A1 and 2B7 and mediated by chemical and enzymatic hydrolysis, respectively^12^. Therefore, aprocitentan can be concomitantly administered with drugs that are inhibitors or inducers of any cytochrome P450 without dose adjustment. In addition, drug-drug interaction studies with midazolam and rosuvastatin showed that aprocitentan does not influence the PK of drugs that are substrates of cytochrome P450 or the efflux transporter breast cancer resistance protein^13,14^.

Aprocitentan may be used to treat patients with resistant hypertension who have comorbidities such as hepatic impairment which may alter the disposition of drugs^15^. In addition, as hepatic metabolism and/or excretion accounted for a substantial portion (i.e., more than 20% of the absorbed drug) of the elimination of aprocitentan, this study was designed to investigate the effect of moderate hepatic function impairment on the PK, safety, and tolerability of 25 mg aprocitentan compared to that of matched healthy subjects.

## Methods

The study (ClinicalTrials.gov: NCT04252495, registration date: 05/02/2020) was conducted at 2 sites, Biokinetica S.A. in Poland and CRS Clinical Research Services Kiel GmbH in Germany, in accordance with the principles of the Declaration of Helsinki and ICH good clinical practice guidelines. The study protocol was approved by the national health authorities of Poland and Germany (Office for Registration of Medicinal Products, Medical Devices and Biocidal Products and The Federal Institute for Drugs and Medical Devices, respectively) and by the Polish Bioethics Appeal Committee and local ethics committee (Ethikkommission bei der Ärztekammer Schleswig–Holstein). Written informed consent was obtained from all subjects prior to any study procedure.

Due to the coronavirus disease 2019 (COVID-19) pandemic, appropriate measures were taken at the sites to protect the subjects and staff members.

### Study design

This was a two-center, open-label, single-dose, Phase 1 study to investigate the PK, safety, and tolerability of a single oral dose of 25 mg aprocitentan in subjects with moderate hepatic impairment classified using the Child–Pugh scoring system (i.e., Child–Pugh B, n = 8) and compare these to healthy subjects (n = 8). Following a screening period of 3–21 days (or 11–21 days for women of childbearing potential) prior to dosing, subjects returned to the clinical facility on Day –1. On Day 1, a single oral dose of 25 mg aprocitentan was administered in the morning to each subject under fasted conditions. A single dose of 25 mg aprocitentan, administered as one tablet containing 25 mg aprocitentan, was selected as it represented the highest dose being investigated in the Phase 3 study. Subjects were discharged on Day 2 after the 24 h post-dose PK blood sample and safety assessments had been performed, unless there was a medical reason for the subject to remain at the clinical facility. Thereafter, all subjects returned to the clinical facility for ambulatory assessments on Days 4, 8, 10, 12, and 14. An end-of-study (EOS) visit took place on Day 14 or 15.

### Study population

Male and female subjects between 30 and 79 years of age (inclusive) with a body mass index (BMI) of 18.0–32.0 kg/m^2^ or 18.0–35.0 kg/m^2^ (applicable for Poland and Germany, respectively) and age-appropriate renal function confirmed by creatinine clearance (according to the Cockcroft and Gault formula^16,17^) were eligible for this study. Women of childbearing potential were required to have negative pregnancy tests at screening and on Day –1 and had to use a reliable method of contraception from screening until 30 days after dosing. Healthy subjects were defined as such based on physical examination, medical history, 12-lead ECG, vital signs, routine hematology, clinical chemistry, coagulation, and urinalysis tests and were matched to subjects with moderate hepatic impairment regarding sex, age (± 5 years), body weight (± 10%), and height (± 10%) at screening. Subjects with moderate hepatic impairment were categorized according to the Child–Pugh classification (Child–Pugh grade B, score: 7–9)^18^. Child–Pugh score was based on screening laboratory test results for serum bilirubin, serum albumin, prothrombin time, and the state of hepatic encephalopathy, with or without ascites.

Women who were pregnant or breastfeeding were not eligible, as were subjects with positive alcohol and drug screening tests, excessive caffeine intake or clinical evidence of any disease or any surgical or medical condition with a potential to interfere with the absorption, distribution, metabolism, or excretion of aprocitentan except for those related to liver impairment (e.g., appendectomy and herniotomy allowed, cholecystectomy not allowed). In addition, subjects with hepatic impairment with acute hepatitis, severe ascites and/or pleural effusion, history of hemorrhagic stroke, recent gastrointestinal bleeding or myocardial infarction, hepatic cancer, primary biliary cirrhosis, or any form of cholestatic disease were excluded from study participation.

Healthy subjects were not allowed to take any concomitant medication except for contraceptives, hormone replacement therapy, and medications for treatment of adverse events (AEs). Subjects with moderate hepatic impairment were required to be on stable concomitant medication for at least 3 weeks prior to screening and were expected to be stable during the study.

### Safety and tolerability

The safety and tolerability of a single dose of aprocitentan were evaluated throughout the study based on reported AEs, results of physical examination, assessment of body weight, vital signs, 12-lead ECGs, and clinical laboratory variables (hematology, clinical chemistry, coagulation, and urinalysis).

### Bioanalysis

Blood samples (approximately 3 mL) for PK assessments were collected in tubes containing ethylenediamine tetra-acetic acid (EDTA) at pre-dose and at 1, 2, 3, 4, 6, 7, 8, 10, 12, 15, 24, 72, 168, 216, 264, and 312 h post-dose. Two blood samples of approximately 4 mL each were collected in tubes containing EDTA to determine the extent of plasma protein binding (PPB) of aprocitentan at 8 and 168 h post-dose. Plasma concentrations of aprocitentan were measured using a validated liquid chromatography method coupled to tandem mass spectrometer^9,19^, with a lower limit of quantification (LOQ) of 5 ng/mL. Calibration and quality control samples were analyzed throughout the study. The inter-batch precision was ≤ 5.4% and the inter-batch accuracy 0.8–5.2%. The PPB of aprocitentan was determined using rapid equilibrium dialysis as previously described^10^.

### Pharmacokinetic analysis

The PK parameters of aprocitentan were obtained as previously described^10^ by non-compartmental analysis using Phoenix WinNonlin version 8.0 (Certara, Princeton, NJ, USA). Both C_max_ and time to reach C_max_ (t_max_) were directly obtained from the measured individual plasma concentrations of aprocitentan. The area under the plasma concentration–time curve (AUC) from time zero to infinity (AUC_0−∞_) was calculated according to the linear trapezoidal rule by combining AUC from zero to time t of the last concentration above the LOQ (AUC_0−t_) and AUC_extra_, where AUC_extra_ represented an extrapolated value obtained by C_t_/λ_z_. C_t_ was the last plasma concentration above the lower LOQ and λ_z_ the terminal elimination rate constant determined by log-linear regression analysis of the measured plasma concentrations of the terminal elimination phase. The t_½_ was calculated as ln2/λ_z_. The apparent clearance was calculated as dose/AUC_0−∞_. The apparent volume of distribution was calculated as dose/λ_z_ × AUC_0−∞_. Aprocitentan plasma concentrations below LOQ were set to zero and included in the calculation of means. PPB of aprocitentan was calculated as 100 − (C_u_/C × 100%), where C_u_/C was the ratio of unbound to total plasma concentration of aprocitentan.

### Statistical analyses

The sample size of 8 subjects per group was based on empirical considerations. Assuming a standard deviation (SD) of 0.24 and 0.23 for log-transformed AUC_0−∞_ and C_max_, respectively, it was estimated that, with a sample size of 8 evaluable subjects per group, the lower and upper bounds of the 90% confidence interval (CI) for the geometric means ratio (GMR; moderate hepatic impairment subjects as test and healthy subjects as reference) would be approximately 0.81 and 1.24 for AUC_0−∞_ and 0.82 and 1.22 for C_max_ if the ratio was 1.

PK parameters of aprocitentan were summarized as done before^10^ using geometric means and their 95% CI, arithmetic means and their 95% CI for PPB, or the median and range values for t_max_. Differences in PK parameters between subjects with moderate hepatic impairment and healthy subjects were explored using their GMRs and 90% CI with healthy subjects as reference. Differences between groups for t_max_ were explored using the Hodges-Lehmann estimate of the median of difference and its 90% CI with healthy subjects as reference. The safety and tolerability data were analyzed descriptively by group. All statistical analyses were performed using SAS® software (version 9.4; SAS Institute, Cary, NC, USA).

### Ethics approval

All procedures performed in this study involving human participants were in accordance with the ethical standards of the institutional and/or national research committee and with the 1964 Helsinki Declaration and its later amendments or comparable ethical standards.

### Consent to participate

Written informed consent was obtained from all individual participants included in the study prior to any study-mandated procedure.

## Results

### Demographics

A total of 17 subjects (8 subjects with moderate hepatic impairment and 9 healthy subjects) were included in the study and received the planned single oral dose of 25 mg aprocitentan. One healthy male subject was excluded from the PK analysis as he prematurely discontinued from the study after Day 4 for personal reasons. The remaining 16 subjects completed the study, and all 17 subjects were included in the safety analysis.

Demographic characteristics were similar across both groups with respect to age, weight, height, and BMI (Table 1). One female was enrolled in each group, and all 17 subjects were White. Subjects with moderate hepatic impairment had a Child–Pugh score of 7 (n = 6) or 8 (n = 2) and they all received concomitant medications at baseline (e.g., diuretics, mineral supplements, and drugs used to treat acid-related disorders) for the treatment of their liver cirrhosis and other ongoing comorbidities. In healthy subjects no medication was ongoing at baseline.

**Table 1:** Demographic characteristics.

	Moderate hepatic impairment (n = 8)	Healthy subjects (n = 9)
Sex (M:F)	7:1	8:1
Age (years)	60.6 (8.4)	61.1 (9.0)
Weight (kg)	80.3 (12.6)	77.9 (11.2)
Height (cm)	174 (5.6)	172 (7.7)
BMI (kg/m^2^)	26.5 (3.8)	26.2 (3.2)
Child–Pugh score*		
Score 7	6 (75.0)	
Score 8	2 (25.0)	

Data are expressed as arithmetic mean (SD), as a ratio for sex, or as n for Child–Pugh score (%).

*BMI* body mass index, *F* female, *M* male, *n* number of subjects, *SD* standard deviation.

*Calculated at screening.

### Pharmacokinetic evaluations

**Figure 1:**
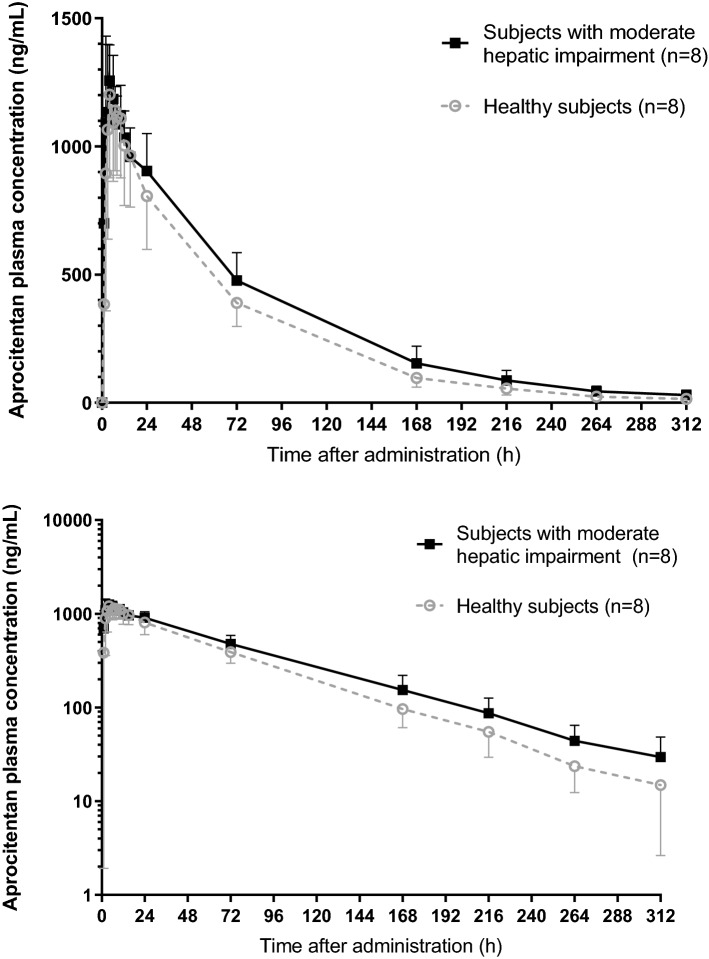
Arithmetic mean (± SD) plasma concentration–time profiles of a single oral dose of 25 mg aprocitentan on a linear (upper) and semilogarithmic scale (lower) in subjects with moderate hepatic impairment and matched healthy subjects.

The plasma concentration–time profiles of aprocitentan were similar between subjects with moderate hepatic impairment and healthy subjects (Fig. 1). Peak plasma concentrations were reached at a median of 4.0 h for both subjects with moderate hepatic impairment and healthy subjects (Table 2). There was no difference between groups in C_max_ as indicated by a GMR of C_max_ (90% CI) of 1.03 (0.86–1.24). After reaching C_max_, aprocitentan concentrations decreased slowly in both groups as indicated by a t½ of 56.4 h and 48.3 h, respectively. The apparent clearance, CL/F, in subjects with moderate hepatic impairment was lower (251 mL/h) compared to that of healthy subjects (309 mL/h). The apparent volume of distribution, V_Z_/F, was similar between groups (20.4 L and 21.5 L in subjects with moderate hepatic impairment and in healthy subjects, respectively). Overall, this resulted in higher exposures in terms of AUC_0−t_ and AUC_0−∞_ of 22% and 23%, respectively. The GMRs and 90% CI of AUC_0−t_ and AUC_0−∞_ were 1.22 (1.02–1.46) and 1.23 (1.02–1.48), respectively. Aprocitentan was highly bound to plasma proteins (mainly albumin) in both groups, with PPB values ranging from 98.7 to 98.9% and from 98.9 to 99.0% at 8 and 168 h after dosing, respectively. All PK parameters are summarized in Table 2.

**Table 2:** Plasma pharmacokinetic parameters of aprocitentan in subjects with moderate hepatic impairment and healthy subjects after administration of a single oral dose of 25 mg aprocitentan.

Parameter (unit)	Moderate hepatic impairment (n = 8)*	Healthy subjects (n = 8)*	GMR^ǂ^	90% CI of GMR^ǂ^
C_max_ (ng/mL)	1307 (1165–1466)	1266 (1024–1566)	1.03	0.86, 1.24
AUC_0−t_ (h × ng/mL)	97,121 (82,383–114,495)	79,758 (66,663–95,424)	1.22	1.02, 1.46
AUC_0−∞_ (h × ng/mL)	99,722 (84,007–118,378)	80,975 (67,705–96,846)	1.23	1.02, 1.48
t_max_ (h)	4.0 (2–7)	4.0 (2–7)	0.0	−2.0, 1.0
t_½_ (h)	56.4 (47.3–67.3)	48.3 (42.7–54.5)	1.17	1.00, 1.37
CL/F (mL/h)	251 (211–298)	309 (258–369)	0.81	0.68, 0.98
V_Z_/F (mL)	20,396 (17,710–23,490)	21,490 (17,728–26,051)	0.95	0.80, 1.13
PPB–8 h (%)	98.7 (98.6, 98.9)	98.9 (98.8, 99.0)	NC	NC
PPB–168 h (%)	98.9 (98.7, 99.1)	99.0 (98.9, 99.1)	NC	NC

*AUC0−∞* area under the plasma concentration–time curve from zero to infinity, *AUC0−t* area under the plasma concentration–time curve from zero to time t, *CI* confidence interval, *CL/F* apparent clearance, *Cmax* maximum plasma concentration, *GMR* geometric means ratio, *n* number of subjects, *NC* not calculated, *PPB* plasma protein binding; *t½* terminal half-life, *tmax* time to reach maximum plasma concentration, *VZ/F* apparent volume of distribution.

*Data are expressed as geometric means (95% CI), as arithmetic means (95% CI) for PPB, or as median (range) for t_max_.

^ǂ^For t_max_, the median of differences and 90% CI are shown.

### Safety and tolerability

The only treatment-emergent AE reported during the study was a headache of mild intensity (subject with moderate hepatic impairment), not considered related to study treatment by the investigator, and that resolved after treatment with an analgesic. No serious AEs (SAEs), AEs leading to study discontinuation, or clinically relevant changes in vital signs, body weight, laboratory variables, and ECG variables were identified during the study. Following single-dose administration of aprocitentan, a transient decrease in hemoglobin, hematocrit, and red blood cell count was observed in subjects with moderate hepatic impairment that was larger than that observed in healthy subjects. In addition, a transient and minor effect of treatment with aprocitentan was observed on the mean and median changes from baseline of systolic and diastolic blood pressure (SBP and DBP, respectively) at 8 h post dose. However, none of the individual values for SBP and DBP outside the normal range were judged to be of clinical relevance by the investigators.

The healthy male subject that prematurely discontinued from the study for personal reasons after Day 4 returned to the clinic for an EOS visit. At this visit (i.e., Day 16) he experienced increased alanine aminotransferase and aspartate aminotransferase levels of approximately 4 × the upper limit of normal that were not classified as clinically relevant by the investigator. The liver enzymes measured at 24 h (Day 2) were decreased compared to baseline. It was suspected that the changes in liver enzymes at EOS resulted from a change in lifestyle and diet (the subject prematurely discontinued from the study). In addition, the subject declined to perform additional tests after EOS. A safety follow-up telephone call 32 days after EOS did not raise any safety concern. The plasma concentrations of this subject measured up to the day of discontinuation were within the range of those observed in other subjects in the group.

## Discussion

This study was conducted to assess the PK, safety, and tolerability of a single oral dose of 25 mg aprocitentan in subjects with moderate hepatic impairment compared to matched healthy subjects.

In healthy subjects, the PK and safety profiles of aprocitentan were in line with those observed in previous studies^9,10,12^. In subjects with moderate hepatic impairment, aprocitentan was similarly absorbed as in healthy subjects, i.e., with t_max_ 4 h. C_max_ was also similar between groups, with 90% CI of the GMR falling entirely within the regulatory acceptance bioequivalence limits. However, exposure to aprocitentan in terms of AUC_0−t_ and AUC_0−∞_ increased on average by 22% and 23%, respectively, in subjects with moderate hepatic impairment compared to healthy subjects. As aprocitentan was confirmed to be safe and well tolerated at multiple doses up to 100 mg^9^, the increase in exposure of < 25% is not considered clinically relevant. The exposure increase in subjects with moderate hepatic impairment was observed together with a small decrease in CL/F compared to healthy subjects. Since V_Z_/F was similar between subjects with moderate hepatic impairment and healthy subjects, the small prolongation in t½ of aprocitentan in subjects with moderate hepatic impairment could be explained by the small decrease in aprocitentan CL/F. The increase in exposure observed in this study is in line with findings in subjects with severe renal impairment and with the elimination routes of aprocitentan^10,12^. As aprocitentan is excreted in both urine and feces, either route of elimination is available.

Hepatic function impairment can lead to a decrease in extent of PPB^15^. However, in this study, PPB was similar between subjects with moderate hepatic impairment and healthy subjects. Aprocitentan was highly bound to plasma proteins (mainly albumin: 98.7–99.0%) in both groups, in line with previously published data in humans^10^.

Administration of a single dose of 25 mg aprocitentan was well tolerated by both subjects with moderate hepatic impairment and healthy subjects, with no clinically significant treatment-related patterns observed for hematology, clinical chemistry, coagulation, urinalysis, vital signs, ECG variables, or body weight. Only one treatment-emergent AE was reported in one subject with moderate hepatic impairment, a headache of mild intensity, in line with observations from previous studies^9,10,13^. The observed transient decrease in hemoglobin, hematocrit, and red blood cell count in this study following single-dose administration of aprocitentan was not considered clinically relevant but was expected based on observations in other studies with aprocitentan and other ERAs^7,9,11,14,20^.

Overall, the PK, safety, and tolerability results suggest that dosing of aprocitentan does not require adjustment in subjects with moderate hepatic impairment. Therefore, these findings can be extrapolated to subjects with mild hepatic impairment, i.e., no dose adjustment is necessary for subjects with mild hepatic impairment.

The limitations of this study are the small number of subjects, the short-term treatment duration (one single dose), and the use of subjects without the target disease indication (resistant hypertension). More safety information after long-term treatment in patients with hepatic impairment and resistant hypertension is needed to assess whether aprocitentan can be safely used in such population. Since subjects with severe hepatic impairment were not studied, it is uncertain if a dose adjustment based on PK of aprocitentan would be necessary for such patient population.

## Conclusion

As patients treated with aprocitentan may have several comorbidities including hepatic impairment, this study was conducted to investigate the PK, safety, and tolerability of aprocitentan in such population. In summary, there were no clinically relevant differences in PK, safety, and tolerability of aprocitentan between subjects with moderate hepatic impairment and healthy subjects after a single dose of 25 mg. Based on these data, aprocitentan can be administered to subjects with mild and moderate hepatic impairment without the need for dose adjustment.

## Data Availability

The datasets generated or analyzed during the current study are available from the corresponding author upon reasonable request.
